# Nurse going native: Language and identity in letters from Africa and the British West Indies

**DOI:** 10.1177/0021989414563149

**Published:** 2015-01-28

**Authors:** Jessica M. Howell

**Affiliations:** Texas A&M University, USA

**Keywords:** African and Caribbean travel writing, colonial medicine, gender and empire, history of science, interwar literature

## Abstract

Colonial nurses were ideal agents of colonial medicine’s supposed beneficence: while practising and teaching “hygiene”, they also reinforced racial and cultural separation. In some cases, however, the nurses took their role as healers and teachers of local populations much more seriously than was authorized implicitly by their employer. This article analyses the circulation of original life writing materials between one nurse, CC, and the Colonial Nursing Association, in order to chart the considerable anxiety around the concept of nurses’ cross-cultural and cross-racial sympathy during the interwar period. I draw upon colonial language studies and women’s travel writing analysis in order to demonstrate that many of these concerns centred on issues of language and communication. By speaking local languages, it was feared that colonial nurses’ loyalty would shift from their employer towards their indigenous patients. This essay places the concept of “going native” within the contexts of nineteenth-century empire literature, racial anthropology and ethnology, in order to suggest that concerns about nurses “going native” were influenced by discourses of degeneration and acclimatization.

Charlie Marlow in Joseph Conrad’s *Heart of Darkness* (1899) explains to his listeners:

The conquest of the earth, which mostly means the taking it away from those who have a different complexion or slightly flatter noses than ourselves, is not a pretty thing when you look into it too much. What redeems it is the idea only. (1988: 10)

Marlow refers to the “idea” or “ideal” of civilization, often used by colonists to justify the exploitation of foreign lands and peoples. Under the auspices of the civilizing mission, European colonialism undertook to save the heathen, educate the ignorant, and heal the sick. Colonial medicine worked to deliver a key part of this mission by supporting indigenous health. However, it often did so by first diagnosing local populations as unhygienic and superstitious: in other words, medically irresponsible. Not only “native” bodies but also local cultures and foreign geographies were found in need of the curative effects of colonial intervention.

Colonial medical discourse worked to “articulate notions of difference” (Vaughan, 1991: 12–13). Warwick Anderson (2006), by examining doctors’ letters, memoirs, and journals, has demonstrated how colonial medicine helped inscribe a “white Australia”, while Megan Vaughan pinpoints the interwar period in Africa, which this paper engages, as a time during which “there was a powerful strand of thinking which saw the erosion of difference as itself a factor which predisposed individuals and groups towards the contraction of certain diseases” (1991: 12–13). From the nineteenth century, medical eugenics had discouraged sexual contact between races, arguing that the resulting mixed-race offspring, or “mulattos”, were weaker or more susceptible to disease (see Josiah C. Nott’s *Types of Mankind; or, Ethnological Researches*, 1854 and F. W. Farrar’s “On Hybridity”, 1864: ccxxii–ccxxix).^1^ There were more subtle ways in which colonial medicine emphasized difference: by recommending that whites continue certain health practices in spite of their change in circumstance, for example, perhaps by keeping up habits of recreation and exercise that protected wellbeing and mental balance against the stressors of colonial life. Not only did these values work to set European subjects apart from local populations; when whites failed to practice civilised health behaviours, such as good hygiene or temperance, this was thought to leave them more susceptible to illness.^2^

Hygiene, temperance, and decorum are commonly associated with the ordered domestic realm; within the colonial context, one of the roles of white women was to uphold domestic propriety, reinforcing such values when they lapsed. Women could serve as administrators’ help mates, sometimes becoming “meliorators of the worst manifestations of indigenous and colonial oppression” (Strobel, 1991: xiii). In many ways, however, colonial nurses were ideal agents of colonial medicine’s supposed beneficence. In the mid-nineteenth century, Florence Nightingale had carefully crafted the image of the “new nurse” as an agent of common sense, propriety, and cleanliness.^3^ Furthermore, as Mary Poovey (1988) has observed, the conceptual links between nursing and empire run very deep. She claims, “With the complacency of an imperialist, Nightingale assumed that bourgeois domesticity and cleanliness were universally desired” (1988: 191). These historical associations authorized the early twentieth-century nurse to serve as an agent of superior European hygiene in the colonial context, as well as an advocate for racial and cultural separatism (Howell et al., 2013). However, in some cases, colonial nurses took their role as healers and teachers much more seriously than was implicitly authorized by their employer.

Philippe Lejeune suggests that life writing scholars perform a kind of “textual genetics”, considering how “life writing is produced as well as what is produced” (2010: 163). The Overseas Nursing Association archive at the Oxford Bodleian Library, encourages this kind of study. The Colonial Nursing Association (CNA, later renamed Overseas Nursing Association or ONA) sent over 8400 nurses abroad between its initiation in 1896 and its termination in 1966. In addition to official memoranda and minutes, the archive contains many individual nurses’ files, which often include their application forms. Sometimes included are notes on the nurses’ appropriateness for overseas service by interviewers; receipts for payments or reimbursements; as well as records of awards they might have received. However, most documents consist of letters written by each nurse to the Association Secretary regarding her experiences serving in colonies, protectorates, and areas of British influence. These life writing materials represent a rich opportunity to explore the complex roles of single, professional women and their relationship to the colonial project. Their analysis allows insight into the networks of censorship and the professional pressure to which nurses were subject, which can shed light on the ways in which discourses of imperial superiority were both enacted and enforced. Also, by comparing successive versions of these women’s letters, one observes in hard copy how a written identity evolves in relation to readership. This essay uses the experiences of one particular nurse, CC, as a case study in order to trace the CNA’s changing engagement with indigenous peoples.^4^

Colonial nurses’ working conditions varied drastically according to location. Some might be sent to fully equipped governmental hospitals, whilst others would find themselves the only European nursing sister in a makeshift clinic, perhaps affiliated with a private mining concern or on the outskirts of a plantation village. The role of the CNA was to promote overseas nursing, recruiting a skilled workforce and then matching nurses to specific job openings. While its work supported the goals of both the Colonial Office and Colonial Medical Service, the CNA was not under the auspices of either, nor could it change colonial policy or medical services directly. Therefore, colonial nurses’ letters provided the Association with crucial information regarding the circumstances of each location.

The letters also created the opportunity for the Association to mould nurses’ behaviour from afar, so that each woman would reflect well on her profession and on her employer (Howell, 2013). The CNA Secretary encouraged positive and adventurous letters, and discouraged through written chastisement any criticism by the nurses of their working conditions (Howell, 2013). She edited and submitted selected letters for publication in professional journals such as *Nursing Notes*, which functioned in turn as a key method of recruitment. The Secretary then also sent copies of *Nursing Notes* to those nurses stationed abroad, creating a feedback loop: nurses read about each other’s triumphs and challenges, but only those deemed appropriate by the Association (Howell, 2013).^5^ The Secretary also censored the nurses’ writing extensively before it was published. When examining the letters in hard copy, one may see the Secretary’s editorial marks pencilled over the nurses’ own handwriting, as she excised those statements she found distasteful before submitting extracts for publication. Therefore, overseas nurses’ writing provides an important example documenting the highly networked and mediated history of discourses of gender, health, and empire.

While a nurse’s relationship to her employer and the circulation of letters between them serves as one important context for studying her writing, so too does the specific geographical, economic, and cultural circumstances of her posts. Bart Moore-Gilbert, in *Postcolonial Life-Writing* (2009), suggests that one take into account the “constitutive importance of geo-cultural location to the formation of identity in western women’s autobiography” (2009: xxi). He asserts that scholars of both women’s autobiography and postcolonial literature should consider the writer’s engagement with “location or dislocation” (2009: xxi). My approach supports these goals by examining the files of individual nurses as case studies and by considering the particularities of the locations where each served. As Gillian Whitlock has established in *The Intimate Empire: Reading Women’s Autobiography* (2000), such a “specific, historical, and contextual reading” offers a corrective to the “idea of a transhistorical female experience, or the notion of the female body as the ground of a unified and consistent meaning” (2000: 3).

To examine how nurses’ identities travel in relationship to their changing circumstances is especially appropriate given the fact that, historically, “Nurses’ … class and gendered, structural place and authority has always made it necessary … to negotiate and form alliances to achieve ends” (Fairman and D’Antonio, 2008: 6). Nurses’ “positionality is always shifting in response to the needs of patients and nurses themselves” (2008: 6). By focusing on nurses’ self-positioning in their letters, one is able to counteract what Amanda Gilroy and W. M. Verhoeven call “The most historically powerful fiction of the letter” — that it functions as a “trope of authenticity and intimacy” without “linguistic, historical, and political mediation” (2000: 1).

Letter writers and readers often have “unequal” positions of “power” (Barton and Hall, 2000: 7). The majority of the nurses’ letters in the archive loosely follow a certain structure, which reflects unequal power between overseas nurses and their employer. The letters are hybrid texts: they function both as reports and memoirs; both as carefully crafted notes to an employer and also as a link to Britain, or home. They bridge what Barton and Hall call the “everyday” and the “official” genres of letter writing (2000: 2–3). Those nurses’ letters written during the early twentieth century often begin with a respectful address, followed by an apology for not writing sooner and an explanation for the delay. The nurse might express thanks for the Secretary’s last letter, or for another small kindness shown. She then outlines the conditions of life and daily work at her post, with perhaps one or two anecdotes or descriptions of travel. Occasionally, she includes a carefully framed request, which could be for a change in post or for information regarding a former colleague. Usually, she concludes with promises to write again soon.

Occasionally, a nurse’s performance while abroad came under review. In these cases, the exchange of letters between nurses and the Secretary provided a venue for the nurse to negotiate or to defend herself. The Secretary, in turn, submitted correspondence regarding contentious cases for the review of the governing Committee of philanthropic ladies who oversaw the Association. The Committee was composed of politicians’ and colonialists’ wives, such as Colonial Secretary Joseph Chamberlain’s wife Mary. A nurse might be relocated or dismissed from her post if either patients or superiors expressed dissatisfaction with her behaviour. In the archive, each nurse’s file contains only those letters and reports that have been preserved and donated by the Association. Therefore, the facts of a nurse’s transgression are often unknown and must be deduced from oblique references in those letters that survive.

CC served abroad in British Honduras and Uganda from 1929 to 1935, at a mid-point between the CNA’s beginning and decolonization, and during a time when the relationships between white Europeans and indigenous peoples were in constant flux. She did not succeed in striking a balance between her desire to serve the needs of local populations and acknowledging the underlying motivation of her employers, for nurses to uphold racial separation and health hierarchies. Both CC’s overseas contracts were terminated, seemingly due to her refusal to cater to the whims of European patients, as well as her troubling affiliation with indigenous nurses and patients.

CC’s repeated complaints regarding her working conditions are not couched in tactful, apologetic or flattering language. Further, she writes as if she has the right to choose the circumstances of her post as well as her duties, in order to support her own personal and professional development. Every letter is peppered with phrases such as “I would like”, “I hope”, “I contemplate”, “I shall decide” or “I shall not decide”, the frequency of which distinguishes her writing from that of her contemporaries. She seems either unaware or unrepentant that her work has been found lacking, despite the fact that her first contract has been terminated, and continues to write as if her own concerns (and her concerns for local populations) are paramount.

CC’s rhetorical self-positioning may show a fundamental miscalculation regarding her constricted role as a professional woman within colonial institutional hierarchies of the 1930s. The CNA sent nurses where there was a need for their labour, and arguably where they would best support ideologies of health and empire — not where they desired to go, where they could learn the most, or even where they believed they could do the most “good”. CC plays an active role in forming discourses of health and empire by attempting to dictate her own professional development, and by explicitly voicing cross-racial sympathy. In her early letters, she repeatedly requests to serve in native rather than European hospitals, thus articulating her “continuing needs and demands as [an] unequal citizen in patriarchal society” (Moore-Gilbert, 2009: xxiii).

Her insistence and self-assertion were not well received. I suggest that by the time her last contract was terminated and she was refused reappointment by the CNA, CC had realised her misjudgment in repeatedly insisting on her right to determine the terms of her own work. Her last letter performs a drastic rhetorical repositioning, as CC re-inscribes herself within traditional discourses of colonial medicine and female susceptibility to the tropical environment. She draws on an ideological system that she believes holds currency for her readers, in order to excuse her behaviour. This is in direct contrast to CC’s earlier letters, in which she implies that she has acclimatized so thoroughly to the tropical heat as to no longer be comfortable in Britain. By the end of her time abroad, she may have intuited that this marked her body in the eyes of her readers as outside British national and racial identity.

What was CC’s unacceptable behaviour? Critics such as Robert Young (1995) have identified both desire and abhorrence towards racial others encoded within colonial discourse. According to Young, in the nineteenth century, colonists’ sympathy for subjects of another race was haunted by the spectre of miscegenation. While it may be initially tempting to read CC’s transgressions as sexual in nature, this essay instead analyses her connection with indigenous cultures in terms of language and translation. Her letters demonstrate a focus on learning the languages of Luganda and Swahili in order to serve local African populations better. CC’s repeated complaints about her European patients and emphasis on communicating with her indigenous patients may have raised concerns with her employer about language acquisition and the risk of going native. Also, CC’s behaviour triggers cultural anxieties about nurses’ over-identification with patients, which is exacerbated when the patients are of a different colour. By contextualizing the concept of “going native” within a history of nineteenth-century empire literature, racial anthropology, and ethnology, I suggest that these concerns were influenced by discourses of degeneration and acclimatization. Rather than “passing” in order to study the locals while maintaining a stable British identity, the worry was that a nurse would become naturalized to her surroundings.

## I. CC and the Association

The stated purpose of the Colonial Nursing Association changed significantly between its initiation in 1896 and termination in 1966. In 1895, Lady Mabel Piggott proposed to Joseph Chamberlain, Colonial Secretary, that the Association should help place British nurses in colonial outposts in order to care for European men and their wives (Colonial Nursing Association Archive 120/1, f 3).^6^ The CNA recruited by depicting overseas nursing as heroic — an opportunity for professional women to take up their portion of the “White Man’s Burden”. In 1920, Lady Piggott described the first two decades of the Association’s work as a grand success. She claimed, “The C.N.A has heard the trumpet-call. It is ready … to stand forth and say to those, flesh of our flesh and bone of our bone, ‘Can we help you?’ Trained women are here, devoted, suitable for pioneer work on the prairies, in the bush, on the homestead” (CNAA 132/3, f 8). The phrase “flesh of our flesh and bone of our bone” suggests that nurses’ role was to maintain a British lineage abroad by healing suffering white bodies. Further, by describing nurses’ labour as “pioneer work on the prairies, in the bush, on the homestead”, Lady Piggott both conflates disparate colonial geographies and erases their local populations. Indeed, during the early years of the CNA, the nurses’ role was explicitly to help recreate civilized standards of medical care abroad and to re-inscribe the boundaries between white and native populations by setting up a space of domestic and social order within the colonial environment (Howell, 2013).

By the middle of the twentieth century, however, the Colonial Nursing Association had been renamed more neutrally the Overseas Nursing Association, and its purpose had adapted along with the changes in colonial policy and global politics. A 1949 pamphlet produced for Guy’s Hospital shows how profound the change had been: “by far the larger part of [the overseas nurse’s] work”, it explains, “will be caring for the people of the country, African, Asiatic, Arab and so on, and training and supervising local nurses and midwives”. The nurse should, “above all, have a sympathetic understanding of the beliefs and customs, often strange and very different from her own, and a very genuine interest in the people among whom she is working”. Her role is “primarily to help the colonial peoples sooner or later to run their own health services”. Such a nurse should have “integrity of character” (CNAA 132/4, f 44).

While the rhetoric of this pamphlet is far from unproblematic (“African, Asiatic, Arab and so on”), one may see reflected in it a gradual change in perception regarding other cultures. For example, a first draft of this pamphlet states that a white overseas nurse may find “much to reward her in the affection and dependence of her (native) nurses”. Miss Gawan Taylor, the author and Secretary of the Overseas Nursing Association during this time, revised this line before publication to read, “Most nurses find their reward in the affection and trust of their nurses and in the widening of their knowledge” (CNAA 132/4, f 61). This change suggests a gradual revision of earlier paternalist (or maternalist, as the case may be) assumptions regarding the gratifying dependence of “dusky races”. The Association did not, however, openly advocate nurses’ “sympathetic understanding of (local) beliefs and customs” until mid-century. CC served abroad during the years preceding the Second World War and well before decolonization. Though trained to support indigenous peoples’ health and told to learn local languages, it seems that she was not expected to attempt substantial cross-cultural understanding.

CC’s first post in British Honduras, 1929–1932, spanned several major upheavals in the colony: with the Great Depression, orders for mahogany and chicle (an ingredient in chewing gum) had decreased and local unemployment had skyrocketed, causing labour unrest and protests. Then a severe hurricane in autumn 1931 destroyed Belize Town, killing hundreds of people and wrecking much of the housing (*The Sunday Times*, 1931). While the economy receives no mention in CC’s letters to the CNA Secretary and the hurricane is referred to only in passing, it is significant that this nurse expresses kindness towards local peoples during a time when they were perceived to threaten British colonial interests through their agitation for workers’ rights.

CC apparently did not suffer from the heat in British Honduras. She claims, “It is very difficult for people from a cooler climate to become + get their babies acclimatized. Once this is done, however, most people find the climate healthy” (CNAA 129, Item 13, f 2 r). With these remarks, CC writes herself as a white subject whose body has successfully adapted to the tropical climate. This is borne out by her letter to the CNA Secretary upon return to England on the 28th of July, 1932. CC writes, “We had a lovely voyage, but a rainy welcome at Liverpool and cold, windy weather here, which made me long for our Belize sunshine. I feel that almost at any cost I must return” (f 4).^7^ The strength of her emotion is striking, as is the fact that her preference has shifted from British to tropical environments.

According to earlier theories of acclimatization, this shift could be interpreted to mean that the nurse’s very body has been colonized by the tropical climate (for more on theories of acclimatization, see Harrison, 1999). Theories regarding the degeneration of white bodies under the influence of extreme heat reverberated in colonial medical discourse from earlier eras through to the early twentieth century.^8^ Whether or not colonists succumbed to lassitude, enervation, or immoral behaviour “under the midday sun”, the long-term effects of tropical heat generally were not considered beneficial. On its own, CC’s comment that she longs for Belize sunshine might not be enough to raise concern — many nurses wondered in their letters how they would ever stand the British winters again after time abroad — but in conjunction with the stress she places on her connection with local peoples, a portrait begins to emerge of a nurse whose loyalty to her own nation and culture might be questioned by her employer.

CC says in a letter from Belize written on 19 June 1932, “I have been comparatively happy here and love the people very much indeed, and for their sakes would like to return” (f 3 r). There is a discontinuity between the CNA Secretary’s notes on CC’s employment file and her own representation of her work abroad. Though the word “terminated” is handwritten in red pen next to the date and location of CC’s post in British Honduras, no indication is given in the nurse’s letters that either she or her employers were dissatisfied with her work in Belize.

CC is decidedly less pleased with her subsequent post in Uganda. Rather than expressing hesitation about working with black Africans, as did many of her colleagues, CC showed curiosity about her forthcoming duties. On the 8th of December 1932, she writes to the Secretary from England, “Can you tell me whether it is the ‘English Hospital’ or the ‘GNH (Government Native Hospital)?’” [at Kampala]. “I do not mind a bit, but one should just like to know”. The Secretary replied right away that there were both European and Native Hospitals near Kampala (f 6 v; reply from Secretary f 7).

However, during her time in Uganda, CC was disappointed to find that she would not be working extensively with indigenous peoples, and she expressed her disappointment repeatedly in letters to the Association. She was posted to the European Hospital, which she writes is just like a “Nursing home” in Britain with no real role teaching or mentoring local nurses. Initially, she hoped that she would work with African populations — on the 27th of March, 1933, she states, “I’ve done very little theatre work so far, but perhaps I shall when I go to the Native Hospital at Mulago”. By the first of September, she begins to express frustration — “I do so miss my Infant Welfare work and my teaching of Midwives etc. Perhaps if I go to an outstation it will be better, but it seems as if it is the same everywhere”. She feels that “by coming out here to nurse our own people”, she and her fellow nurses are not doing enough to directly help the local population: “it does seem so passive”. She says, “I want to be *active*” in teaching black Africans (f 9; Emphasis in original).

By the next summer, 1934, CC was feeling restive. She writes:

I went to Mulago, the Native Hospital for 3 months, but … I only did 3 months + have been sent back here again. I … did so long to stay there + do some Junior teaching work. However, I am rather puzzled as to what is intended for me. European work is not easy for me. (f 13)

CC found her “hopes of real work among the Africans” thwarted, however, and finally seemed to alienate her European patients and superiors. She says:

Many Europeans have appreciated my work in E.H. so I am told, but I am afraid I am unfortunate in ‘falling foul’ (if I may so express it) with a certain type of people who demand insincerity and exaggeration of their ills. This type usually seems to gain the ear of the ‘Powers that be’. (f 18)

CC’s contract in Uganda was terminated early. A handwritten note by the Secretary underscores her rejection — “Not recommended for re-engagement” and on the front of her file she is deemed “unsuitable”. While the exact reasons behind CC’s “unsuitability” may remain a mystery due to sparse biographical information and her incomplete file, some compelling possibilities arise from her various forms of identification with local populations.

## II. A new mother tongue

Bronislaw Malinowski first defined “going native” as it applies to anthropology and ethnology, based on his experience living in New Guinea from 1914 to 1918. He suggested that “‘to grasp the native’s point of view, his relations to life, to realize his vision of his world’” anthropologists should take on the role of participants rather than observers in order to best pursue the “‘study of native peoples and cultures’” (quoted by Kanuha, 2000: 439). As observed by V. K. Kanuha, Malinowski’s command to “go native” was very much of the period in which he wrote, a time when there was a clear division between mainstream (white, male, heterosexual, Western) society and the non-Western world “occupied by the objects of science commonly known as ‘natives’ and ‘savages’” (2000: 441). In other words, the concept of going native presupposes a strict division between self and other, white and nonwhite.

Arguably, this concept has stayed much the same since the era of Malinowski’s studies. In *The Structure of Scientific Revolutions* (1970), Thomas Kuhn defines going native in terms of an epistemological shift, made possible by a shift in language. He says:

To translate a theory or worldview into one’s own language is not to make it one’s own. For that one must go native, discover that one is thinking and working in, not simply translating out of, a language that was previously foreign. (1970: 204)

Not only to speak but also to think in another language, then, becomes a prerequisite for cross-cultural understanding. The influence of anthropological thinking upon the philosophy of science is evident in the excerpt from Kuhn. Much as anthropology makes its “other” and creates for this “other” a separate time and space, the concept of a scientific paradigm shift is also dependent on temporal divisions (“what I think now” versus “what I thought before” and indeed “what I will think in the future”). The contrast is between a currently held theory (“self”) and a new or foreign theory (“other”).

While Kuhn’s and Malinowski’s work give a positive cast to going native, believing this to be the only manner by which one may understand other peoples or ideas, both men seem to assume that the self can always return to its previous state — the anthropologist can cease to participate in native rituals and the scientist could revisit his or her first scientific “language”. However, the antecedents of the concept of going native reach much further back into discourses of degeneracy, and reflect a very real concern that there could be no going back. To go native was seen in the nineteenth century, not just to step away from one’s own people but also to take a step back into savagery. Stephen Arata asserts that “‘degeneration’ had no single fixed meaning or material referent”, but that the word could apply “culturally, biologically, and in other contexts” (1996: 174). As is evident from discourses of acclimatization, empire literature was shot through with the worry that the tropical environment would corrupt both imperialists’ health and their cultural values.

The Victorian worry that individual imperialists would succumb to the influence of life abroad was always also a worry that “The imperial race itself (would fall) prey to a collective form of going native” (Brantlinger, 2011: 67–8). The Victorians struggled with a concern that passing as a native temporarily could cause one to permanently change racial and cultural allegiance. Writers performed control over the emotional component of this process by maintaining a thin veneer of professionalism — by claiming that they sought to study rather than to join the local populations.

For example, British explorer Sir Richard Burton became famous by publishing his narrative of going native as the character Abdullah, in *A Personal Narrative of a Pilgrimage to Al-Madinah and Meccah* (1855). Burton’s extensive knowledge of foreign languages allowed him insight into other cultures. However, as Ben Grant rightly points out, Burton’s very perception of that other culture was coloured by his Western standpoint. When Burton arrives in Alexandria, for example, he describes the atmosphere with the word “Sayf”, which he says is “‘untranslatable in our mother tongue’”. However, he then proceeds to attempt to translate it as “‘The savouring of animal existence; the passive enjoyment of mere sense; the pleasant languor, the dreamy tranquility, the airy castle-building’”. “Sayf”, in this case, becomes “synonymous with the Orient itself, as that which distinguishes this place from Europe”, but as Grant observes, it is still defined in terms of Burton’s own associations with the exoticism of the East (2009: 60). In other words, though he may be using the term accurately, Burton chooses it as a Western subject, because of the contrast it offers with his usual experience. While Grant implies that Burton is unaware of continuing to filter the world around him through his European subjectivity, in fact Burton here may be cannily performing for his audience his stable identity as an observer. Arguably, Malinowski and Kuhn both implicitly find comfort in the idea that one may always recover a state of civilized behaviour or go back to a familiar theory.

However, in the nineteenth century it was believed that not all elements of going native were under one’s control. The reason why his performance of going native excited and titillated Burton’s readers to such an extent may have been because he was walking a tightrope in front of their eyes. Tropical climates, it was thought, could wreak physical as well as mental and emotional changes upon Europeans that rendered them “other” than when they began their journey. Perhaps the emblematic character who goes native, in this sense, is Mr Kurtz in *Heart of Darkness*. Both his understanding of the local African language as well as his physical symptoms of illness are both very much linked to his process of going native.

As Marlow is bringing Kurtz back to civilization from the wilderness of Africa, the villagers pursue the party to the steamship. Kurtz’s African mistress rushes out “to the very brink of the stream. She put out her hands, shouted something, and all that wild mob took up the shout in a roaring chorus of *articulated*, rapid, breathless utterance” (emphasis added). Marlow asks Kurtz, “‘Do you understand this?’” and Kurtz looks past Marlow with “fiery, longing eyes, with a mingled expression of wistfulness and hate. ‘Do I not?’ he said slowly, gasping, as if the words had been torn out of him by a supernatural power” (Conrad, 1899/1988: 145). Kurtz’s connection to the villagers is very much linked to his speaking, and understanding, their language. Further, the character of Kurtz is not retrievable, either physically or ideologically. He cannot be brought back by boat, due to his compromised health, and he can never recover the subjectivity of a confident and complacent imperialist.

Perhaps unsurprisingly, due to the constraints of gender norms, female nurses were given much less respect for transgressing boundaries than famous male explorers. Rather than believing women would be able to maintain professional distance alongside sympathy for their patients, the Association assumed that nurses were in danger of succumbing to the lure of the tropics. The case of CC implies that, once a nurse crossed the boundaries of language and racial sympathy, she was considered permanently tainted.

Though nurses in the 1930s were encouraged to gain a working knowledge of their patients’ languages, this seems to have been more to maintain order and pursue effective training than to foster mutual sympathy. As an example, an article “Nursing in Tanganyika Territory” in the September 1930 edition of *Nursing Times* reads,

For any kind of nursing in Tanganyika, one should know the language of the Africans. Swahili is not a difficult tongue, especially if one has studied its grammar before coming out. … It is worth remembering that Africans have among themselves a very high standard of good manners, and that although they may obey, they will not respect those who trespass against it. Above all they honour one who is *taritibu*, that is, calm under any circumstances; to lose one’s head or one’s temper is, in African eyes, to lose one’s self-respect. (1930: 1069)

As with Burton’s translation of “Sayf”, one observes how Western subjects select concepts that either reinforce or contrast in interesting ways with their own value system. It seems no accident, then, that this author offers a superficial translation of “*taritibu*”, an African word that reinforces dignified behaviour in the nursing sisters.

Further, in *The Journal of Careers*, February 1934, the Association stresses that goodwill between nurses and indigenous peoples is desirable mainly in that it furthers order in the colonial system of healthcare. The article reads, “Natives require, for their successful handling, firmness, strict justice, no favouritism, and truthfulness, and much tact” (CNAA 132/4, f 81 v). If she is successful, then “gradually, by patience, and by never losing her self-control, [the nurse] can educate these natives to a better mode of life, and an understanding of the elementary laws of hygiene and health” (CNAA 132/4, f 81 r). The “successful handling” of natives recalls Foucault’s description in *Discipline and Punish* (1977) of the function of the naval hospital, which he argues “must be a filter, a mechanism that pins down and partitions; it must provide a hold over this whole mobile, swarming mass” (1991: 144).

CC differentiated herself from her colleagues by showing much more than a passing interest in learning the languages of Uganda. She says:

I worked hard at the Native language — Luganda, believing it would help me to get to Native work. I was sent for three months to the Native Hospital and then back here!! Nothing daunted, I set to work on Swahili, which a new standing order ordained us to learn — still hoping that I should at least be tried at an outstation, for I love the natives, and know I should be successful if I could do the work I am suited for. (CNAA 129 Item 13, f 17)

In fact, nearly every letter in CC’s file has some mention of her extraordinary effort learning languages and her pride in being able to communicate with Africans. She says that, even though she was stationed at the Native Hospital for only a short time, she took “every possible opportunity to see Native life and conditions + have really spent a very useful time out here” (f 21). The question arises, useful to whom? CC’s experience might have been useful to her own professional development. Also, she no doubt assumes, “with the complacency of an imperialist” (Poovey), that her service was useful to the wellbeing of her patients. Finally, her letters and reports contribute to an archive of official discourse, and are thereby “useful” in that they add to the knowledge base of British colonialism regarding “native life” and conditions.

To understand why CC’s study of Luganda and Swahili might have counted against her rather than in her favour, one must take a step back to consider the role of language study as a tool of colonial power. As critics of colonial linguistics and translation studies have observed, from missionaries’ earliest contact with indigenous peoples to the first attempts by European traders to exploit foreign resources, learning the local language has been recognized as a potent tool of power and control. As Rachel Gilmour asserts, “The development of colonial linguistics” was “fundamental to strategies by which Westerners interpreted the world, categorized its peoples, and affirmed the superiority of their own position within it” (2006: 2).

The tools of colonial linguistics, which included “grammars, dictionaries, wordlists, reading books” and “philological treatises”, also mapped social hierarchies and power structures, as well as dictated the limits of appropriate contact between races and cultures. Europeans acquired but also stabilized, consolidated and overwrote local languages, in order to make the project of colonial administration more efficient and to suppress ethnic multiplicity. Swahili was perhaps the emblematic case of such linguistic domination: as Joseph Errington and Johannes Fabian have documented, from the nineteenth century, Swahili became an object of “descriptive, codifying attention because of [its] growing salience for regimes that progressively penetrated territories and communities” (Errington, 2001: 29). After the First World War, the goals of British Empire shifted from “safeguarding of routes” to the “administration of territories”, and Swahili assisted in the “assemblage” of a “large, stable labor force” (Fabian, 1991: 42–3).

One of the reasons that missionaries and colonists stressed learning local languages was because it made teaching English easier, and thereby facilitated the spread of “civilization”. However, the goal of acquiring African languages touched a live wire of ambivalence regarding over-identification with colonized peoples. Learning the local language often meant having extensive contact with local peoples, who explained concepts and terms to white subjects: it was worried that this contact could inspire in white Europeans doubts regarding their own superiority. Furthermore, by the 1920s, Africans had made great strides in English literacy; they were gaining more and more semi-skilled and skilled employment; they were even opening their own evangelical churches. In response, Britain instituted the so-called “native policy”: “African ‘otherness’ was shifting from being a difficulty (to be overcome) to being a policy (to be upheld)” (Jeater, 2001: 459). For example, within the South African context, the Native Affairs Department began to discourage contact between native translators and colonial administrators. They discouraged cultural exchange and encouraged “languages that were for talking *at* Africans, not *with* them” (2001: 468; Emphasis in original).

Those who still took seriously their responsibility to converse with local subjects were perceived with increasing suspicion. During the period between 1925 and 1935, when CC was serving abroad, there was a “flurry of activity around vernacular languages” (460). The colonial administration began viewing with distrust those Europeans who could converse with locals, and perhaps had become “too close to ‘the natives’” (460). As Errington claims, “Languages became targets for anxieties projected out of contradictory demands of pragmatic colonial policy on one hand, and ideas about linguistic identity on the other” (2001: 30).

The Overseas Nursing Association’s stated goal in 1930 was to “train the natives themselves to take on an ever increasing part in filling in such service any posts for which individuals may increasingly become qualified” (Solano and Rafferty, 2007: 1056). According to this aim, CC should have been an ideal candidate for Overseas Nursing. It was perhaps her feared over-identification with patients that raised concerns in her employers. As Mary Poovey (1988) has demonstrated, Florence Nightingale’s mid-nineteenth-century reformers worked to neutralize concerns about nurses becoming too emotionally involved with patients and transgressing the boundaries of propriety. The “new nurse” was meant to be sympathetic but detached, attentive but brisk and professional, handling any resistance on the part of the patient as if he or she was a recalcitrant child (Nightingale, 1860). Over-identification with the patient could compromise the nurse’s reputation as a woman, and the reputation of nursing as a profession. In the case of CC and her colleagues, it could also compromise colonial authority and social order.

However, both nursing and colonial discourses also draw heavily upon the discourses of charitable or Christian love. Though one cannot be sure of the forms and structures of CC’s love for the natives, or how it was interpreted by her employers, by saying that she wanted to assist local peoples and serve them, she entered in to a long-standing tradition of selfless love in the service of God and nation.^9^ CC’s love could have simply been meant to indicate “sympathy … concern for the other’s welfare” (“Love”, n.d.: 2a). However, even though she may be casting her service as a medical mission, CC justifies and perpetuates colonial intervention in indigenous health practices. In this case, it seems that her employer simply privileged the love of propriety and national interest over the love of service.

CC’s contract in Kampala was terminated early: she returned in April 1935 to England rather than in July as scheduled. The last letter in her file is written from Kampala. It is addressed to Lady Antrobus and Mrs Harrington directly, two members of the governing Committee. It seems as though there may have been contention regarding her dismissal, as CC says that she had been waiting to write, “hoping against hope that Mrs. Harrington’s wish might have been realised and I might have won through to continued service here” (CNAA 129 Item 13, f 17). In this final exchange of letters with the Association, she simultaneously attempts to excuse her failings in Africa as well as to lay the groundwork for future employment. CC seems unaware that she has been deemed “unsuitable”, preferring instead to write as though the work was unsuited for her. She explains her plans to complete a public health course in England, which she hopes will allow her to be reappointed to a role she likes better.

However, the Association soundly rebuffs this continuing insistence. On the 3rd of May, 1935, the Secretary replies:

The Committee are sorry to hear that you have not found your life and work in Uganda satisfactory. They are interested to know that you are proposing to take the Public Health course and hope that you may like this. The Committee feel, however, that it would not be fair to leave you under any impression that the Association will be able to offer you further appointment overseas. They send you their best wishes and hope you may find congenial work in this country. (f 22)

The bland politeness of this letter nonetheless leaves no room for misunderstanding: the Association no longer considers CC employable. Fault is deflected onto the nurse herself. The Secretary avoids making reference to any other contributing factors for CC’s dismissal, saying instead that the Committee “regrets” that the nurse has “not found” her “life and work in Uganda satisfying”. Arguably, the Secretary is using CC’s own language against her, similarly framing work in terms of choice and preference when terminating her employment. Words such as “hope”, “fair”, and “congenial” craft a beneficent persona for the collective Committee, which also speaks with absolute authority.

In her final reply, CC still writes as though she can choose her own terms of employment, but now refocuses her efforts on the possibility of returning to her earlier post, in Belize: “If I do not come back here [Uganda] to native work, with some teaching and scope for public health, I’d like to go back to Belize when Miss Roberts retires” (f 23). However, she also employs discourses of the dangerous tropics in order to defend against the Committee’s rejection. CC senses that her sympathies for the local populations, and her difficulty working with European patients, have been perceived as a sign of racial and cultural disloyalty. In response, she attempts to show she can conform by adopting certain traditional colonialist tropes.

She says that her “first mistake was, I suppose, coming abroad after such a short holiday at home” (presumably between her time in Belize and Uganda). Although from her initial letters to the Secretary it seems she enjoyed excellent health while abroad, she continues, “I trust the Committee will realize my position, which I have to admit was due partly to going abroad too soon after my experience in British Honduras, and partly I am beginning to believe to a temperamental unsuitability to climate, altitude and conditions — though I tried hard to fight against this” (CNAA 129 Item 13, f 23). CC paints a portrait of her Anglo constitution as under threat from the colonial environment. By saying her holiday in England was too brief, CC implies that she did not adequately experience the tempering influence of a more moderate climate. As Kurtz in *Heart of Darkness* illustrates, the “supernatural power” of the jungle could be blamed for any number of colonial transgressions, including going native. Though CC invokes the traditional rubric of degeneration in the colonial tropics, she does so tactically, specifically associating the trouble with the “climate, altitude and conditions” of Uganda. She thereby tries to create the opportunity to return to Belize (which has a different climate and altitude, and so is less dangerous to her constitution).

This final letter highlights the longevity of “climate” as a construct to explain imperial failures (Howell, 2014). The exchange between CC and her employer also demonstrates the mediated nature of colonial discourses of gender, health, and empire. By studying the writing of a specific individual involved in colonial health care, one can observe that early twentieth-century discourses of racial and cultural difference were encouraged and enforced by both rhetorical as well as material means, as CC was subject to both written recrimination and termination of her employment when she did not obey these boundaries. Further, the case of CC demonstrates that colonial workers deployed stereotypes of feminine as well as Anglo constitutional weakness with multivalent purposes, including self-defence. In CC’s final exchange with the Colonial Nursing Association, one observes a professional woman using discourse of health and “the tropics” in order to position and reposition herself within the networks of colonial power structures. CC’s last appeal is the final document in the archive: no answer from the Association is included. Therefore, the case study of CC also demonstrates how adept these power structures were, as late as the 1930s, at rebuffing women’s attempts to use life writing as one method through which to claim professional and personal agency.

## Notes

1. Theories from these texts and others were used to bolster the values of colonial medicine. Sir Francis Galton initiated eugenics as a field in the 1880s, practices that culminated in the extreme philosophies of Nazi Germany.

2. For example, in *Alone in West Africa* (1911), Australian novelist and travel writer Mary Gaunt claims that “A sane and sober life in the open air day and night” is “a more certain preventive against fever than all the quinine and mosquito-proof rooms that were ever dreamt of”. She says that the antidote for the “reckless man” is “quiet, brave, sensible women” (1911: 391) (see Howell et al., 2013). (For more on hygienic practice and racial and cultural boundaries in the colonies, see Bashford, 2004.)

3. Nightingale set out the “new nurse’s” roles, responsibilities and desirable characteristics in *Notes on Nursing: What it is, and What it is Not* (1860).

4. I use an abbreviation of the nurse’s name due to the sensitive nature of the materials in her file. From biographical research, I have ascertained that she is deceased.

5. *Nursing Notes* was “launched independently by editor and proprietor, Emma Brierly” in 1888. The Royal College of Midwives (formally known as the Midwives Institute) then edited the journal in-house. It was produced by the Women’s Printing Society Ltd, which also printed such items as the Women’s Liberal Federation annual reports. At the beginning of the twentieth century, “Nursing Notes” was “the principle means the organisation employed to communicate with nurses and midwives across the country” (see http://archiveshub.ac.uk/data/gb1538-rcm.html?page=3). The journal had a distinct political persuasion — historically, it had campaigned for women’s suffrage and worker’s rights. In 1908 the journal’s name was changed to “Nursing Notes and Midwives Chronicle”, “signifying how it had become increasingly concerned with issues primarily concerning the midwifery profession” (see http://archiveshub.ac.uk/data/gb1538-rcm.html?page=3).

6. Archival material will be cited as follows: box number/number within series of boxes, then folio page number such as “f 8” with “verso” or “recto” indicated by “v” or “r” if the page is double-sided, or simply 8 if the material is printed. All materials are from the Colonial Nursing Association Archive, Mss Brit Emp s400 (CNAA), Bodleian Library of Commonwealth & African Studies, University of Oxford.

7. As all letters cited from CC are to be found in the same Box and File, only folio numbers will be cited after the first full reference.

8. Though the “enervation” suffered by whites in tropical climates took many forms in early twentieth-century medical discourse, the most relevant diagnosis is “tropical neurasthenia”. So named by Charles Woodruff (1905), as Dane Kennedy, Anna Crozier and others have observed, the diagnosis persisted well into the twentieth century (see Kennedy, 1990: 118–40 and Crozier, 2009: 518–48).

9. This love is defined as “regard and consideration of one human being towards another prompted by a sense of a common relationship to God” (www.oed.com) and was in common usage during the nineteenth and twentieth centuries.
